# Pharmacogenetics of Chronic Pain and Its Treatment

**DOI:** 10.1155/2013/864319

**Published:** 2013-05-20

**Authors:** Svatopluk Světlík, Karolína Hronová, Hana Bakhouche, Olga Matoušková, Ondřej Slanař

**Affiliations:** Institute of Pharmacology, First Faculty of Medicine, Charles University Prague, Albertov 4, 12800 Prague, Czech Republic

## Abstract

This paper reviews the impact of genetic variability of drug metabolizing enzymes, transporters, receptors, and pathways involved in chronic pain perception on the efficacy and safety of analgesics and other drugs used for chronic pain treatment. Several candidate genes have been identified in the literature, while there is usually only limited clinical evidence substantiating for the penetration of the testing for these candidate biomarkers into the clinical practice. Further, the pain-perception regulation and modulation are still not fully understood, and thus more complex knowledge of genetic and epigenetic background for analgesia will be needed prior to the clinical use of the candidate genetic biomarkers.

## 1. Introduction

It is well recognized that pain perception as well as pain relief after analgesic treatment display, large interindividual variability in the population that affects selection of analgesics and their dosing in the population. Age, gender, ethnicity, and actual level of stress, mood, or diseases may modify individual pain perception. This alters also the response to drug treatment, which represents a complex interaction between analgesic medication and organism. Several mechanisms may be involved in the pain relief either as drug targets or as drug metabolizing enzymes/transporters, and the genetic variability in these processes influence the analgesic efficacy in individual patients. This review is focused on highlighting the genetic variability reported to affect chronic pain treatment efficacy. This paper does not provide exhaustive list of polymorphisms reported but focuses on the current status of the most recognized pharmacogenetic areas and variables in the treatment of chronic pain. 

## 2. Neurotransmitters

At least 100 substances can act as neurotransmitters, some of them being released after stimulation of sensory receptors, for example, catecholamines, GABA, and serotonin. Genes associated with synthesis, release, or target proteins for these pain neurotransmitters all represent candidate genes for chronic pain treatment variability. Variation in these pain-associated genes may result not only in variable pain perception but also in variable drug efficacy. Chronic pain and its association with gene polymorphism involved in neurotransmission have been widely studied in animal models. So far, 371 candidate genes have been identified in mice (http://www.jbldesign.com/jmogil/enter.html, accessed in December 2012), and some of them have been also shown to be of clinical relevance for man. Overview of major recently studied pain-associated genes in humans is presented in Table 1. However, the clinical data suggesting possible routine use of all these genetic biomarkers is unconvincing. Studies describing an association of various neurotransmission-related gene polymorphisms with variability of drug response in the treatment of chronic pain are listed in Table 2. 

Recently, Klepstad et al. analyzed 112 SNPs in 25 candidate genes involved in opioid neurotransmission (OPRM1, OPRD1, OPRK1, ARRB2, GNAZ, HINT1, Stat6, ABCB1, COMT, HRH1, ADRA2A, MC1R, TACR1, GCH1, DRD2, DRD3, HTR3A, HTR3B, HTR2A, HTR3C, HTR3D, HTR3E, HTR1, and CNR1) in a large cohort of oncologic patients [22]. No association of these SNPs with opioid dosing (oxycodone, morphine, and fentanyl) was observed. However, haplotypes were not analyzed in this study. Laugsand et al. analyzed 96 single-nucleotide polymorphisms (SNPs) in 16 candidate genes related to opioid or nausea/vomiting signaling pathways (ABCB1, OPRM1, OPRK1, ARRB2, STAT6, COMT, CHRM3, CHRM5, HRH1, DRD2, DRD3, TACR1, HTR3A, HTR3B, HTR3C, and CNR1) for the association with nausea and vomiting in the same cohort of cancer patients. Totally 8 SNPs in 3 genes, COMT, HTR3B, CHRM3 (rs1176744, rs3782025, rs1672717, rs165722, rs4680, rs4633, rs10802789, rs685550), were significantly associated with the interindividual differences in nausea and vomiting among cancer patients treated with opioids [21]. 

Two candidate genes have been clinically studied most widely so far (OPRM1 and COMT).

### 2.1. OPRM1

The *μ*-opioid receptor gene, OPRM1, is the most widely studied gene in association with different aspects of chronic pain. Probable effect of its polymorphism A118G (rs1799971, Asn-40 → Asp) is recognized. In 1998, Bond et al. demonstrated that Asp substitution on the extracellular N-terminal of the receptor determines the same binding affinity for endo- and exogenous opioids (morphine, fentanyl, methadone, naloxone, and met- and leu-enkephalins) with one exception; *β*-endorphin showed higher affinity to the receptor in the 118G variant carriers [68]. This finding allowed the authors to propose a hypothesis that there is a possible connection between the allele and addiction. Somewhat later, Zhang et al. found a 2-fold higher expression of *μ*-opioid receptor in brains of 118G heterozygotes [69]. In the study by Oertel et al., a significant reduction in effectivity of subsequent signaling pathways after the binding of a specific agonist DAMGO was observed. Rate of G-protein coupling in carriers of the G allele reached only 57% in comparison with AA homozygotes [70]. Recently, 4-fold increase in inhibition of Ca channels in the carriers of G-allele was also demonstrated [71]. Contrary to the preclinical data, the results of conducted clinical trials provide unconvincing evidence only. Recent meta-analysis and in particular large-scale cohort study found no evidence for an effect of this polymorphism on opioid dose (oxycodone, morphine, and fentanyl) in oncologic patients [22], although less frequent nausea and vomiting were associated with the polymorphism in the meta-analysis [72]. 

### 2.2. COMT

*Catechol O-methyl transferase* plays a central role in extracellular inactivation of catecholamine neurotransmitters, including dopamine and norepinephrine, in the central nervous system. Val158Met variant (rs4680, G1947A) showed higher enzymatic activity compared to wild type in postmortem human brains [113]. It is associated with a three-to-four-fold variation in COMT enzyme activity and also with individual variation in COMT thermal instability. Lower dopamine levels in carriers of this polymorphism were associated with lower levels of enkephalins in animal models, which in turn lead to downregulation of *μ*-opioid receptor [114, 115]. However, the clinical relevance of these findings is still questionable. Polymorphism rs4680 did not result in variable opioid dosing in the treatment of pain in oncologic patients [22]. Meta-analysis studying rs4680 in different types of chronic pain demonstrated that fibromyalgia or chronic widespread pain could be influenced by the presence of the variant allele. No association was observed with migraineous headache or chronic musculoskeletal pain conditions. According to systematic review of the literature, low COMT activity enhances opioid analgesia and adverse effects in some cancer pains via increasing the absolute amount of opioid receptors [116]. Reyes-Gibby et al. observed significantly lower doses of morphine in cancer patients, carriers of OPRM1 118AA and COMT rs4680 met/met (*P* < 0.012) [23]. 

Three haplotypes (containing alleles rs6269, rs4633, rs4818, and rs4680) which accounted for 96% of all haplotypes observed in the Caucasian population have been identified [79, 117]. Some haplotypes were associated with different phenotypes: low-pain sensitivity, average-pain sensitivity, and high-pain sensitivity, while the risk of developing temporomandibular disorder could be predicted by a single haplotype in this study [79]. Surprisingly, both the low- and high-pain sensitivity-associated haplotypes possessed the val variant of rs4680. According to Nackley et al., interaction of rs4680 with other SNPs (silent mutations: rs6269, rs4633, and rs4818) determines the changes in the secondary structure of the messenger RNA, and these may modify the protein translation and the real *in vivo* activity of the enzyme [118]. The average-pain sensitivity-associated haplotype was modestly associated with greater improvement on a long-term VAS 1 year after lumbar surgery in patients with disc herniation [8]. In another recent study, high-pain sensitivity associated haplotype was associated with moderate or severe headache and moderate or severe dizziness in patients after motor vehicle collision [119]. However, Nicholl et al. found no correlation between COMT “pain sensitivity” haplotypes (rs6269, rs4633, rs4818 and rs4680 alleles) and chronic wide spread pain in two case-control studies (cases *n*_1_ = 164, *n*_2_ = 172; controls *n*_1_ = 204, *n*_2_ = 935) [120]. 

### 2.3. Candidate Genes in New Drug Development

With regard to the preclinical studies, TPRV1 gene product (transient receptor potential cation channel, subfamily V, member 1) appears to be the most promising as a potential target for therapeutic intervention. It is a polymodal nociceptor, the expression of which is upregulated in several painful disorders. Analysis of its function (including knockout mice) revealed that it plays a crucial role in integrating multiple painful stimuli in chronic pain conditions [121]. *TRPV1* gene polymorphism might be an underlying cause of the intersubject variability in pain sensation and response to TRPV1 antagonists [15]. TRPV1 antagonists are undergoing clinical trials in patients with chronic pain at present (reviewed in [122]).

## 3. Proinflammatory Cytokines

Peripheral nociceptors are sensitized by proinflammatory cytokines that are produced by inflammatory cells (CD4+ and CD8+ T cells) in response to disease as cancer or its treatment, that is, cytostatics. This is one of the direct mechanisms leading to hyperalgesia in chronic diseases [123]. Therefore, the research attributed to the polymorphisms in genes coding for these cytokines and their relationship to various diseases including pain and its treatment arises [124–129]. 

Summary of known implications of genetic variability in genes for proinflammatory cytokines is given in Table 3.

### 3.1. TNF-*α*

 TNF-*α* is known to contribute to hyperalgesia associated with chronic illness. After administration of bacterial endotoxin, hyperalgesia can be blocked by functional antagonists of TNF-*α*, for example, TNF-*α* binding protein [130]. Deletion of the tumor necrosis factor receptor type 2 (TNFR2) gene attenuated heat hyperalgesia in tumor-bearing mice, whereas TNFR1 gene deletion played only a minor role [36]. There are few clinical trials suggesting that TNF *α*-308 G/A (rs1800629) is associated with chronic pain perception and treatment success. Variant alleles in TNF *α*-308 G/A were significantly associated with higher pain severity in a study with 140 Caucasians newly diagnosed with nonsmall cell lung cancer [37, 38], and this has been confirmed in another study in newly diagnosed non-Hispanic Caucasian lung cancer patients (*n* = 667) [39]. Higher chance for success of anti-inflammatory phytotherapy treatment in TNF *α*-308 wild-type allele carriers with chronic pelvic pain syndrome has been also proposed in a small study [40].

### 3.2. LTA

Lymphotoxin-alpha (LTA), also known as TNF-*β*, as a member of TNF family is also an important inflammatory marker. In a recent study with lung cancer patients, variant allele in rs5275 was associated with lower pain scores in patients surviving for more than 5 years [41].

### 3.3. COX-2

Cyclooxygenase 2, a product of prostaglandin-endoperoxide synthase 2 (PTGS2), is an enzyme responsible for the production of prostaglandins and represents the target for NSAIDs. As such, it plays a significant role in inflammation and chronic, particularly cancer, pain [131, 132].

Two recent studies reported an association between PTGS2 polymorphisms and pain. In study [39], CC genotypes for rs5275 were at lower risk for severe pain. Close SNP rs5277 was found to predict pain intensity in 1149 Caucasian lung cancer patients in the Mayo Clinic Lung Cancer Epidemiology Project. People carrying one or two minor (G) alleles reported higher pain scores [41].

### 3.4. IL-1

IL-1 is a family of 11 members produced during neuropathic pain and inflammation [133, 134]. Its involvement with pain mediation is undisputable as its, IL-1*β* to be precise, intrathecal injection produces hyperalgesia [135, 136].

Polymorphisms in their genes, as well as in genes of their receptor,have been reported to affect nociceptive response. It was shown that the IL-1*α* (C889–T) (rs1800587) and IL-1*β* (C3954–T) (rs1143634) [137, 138] polymorphisms, and an 86-base pair repeat (VNTR polymorphism) in the IL-1 receptor antagonist (IL-1Ra) [139] genes are associated with the regulation of the IL-1 and IL-1Ra production. Association between IL-1*α* (C889–T) polymorphism with pain intensity was revealed in study in Finnish men (*n* = 1832) with low back pain. Moreover, it was implicated that IL-1*β* (C3954–T) and the IL-1RN (G1812–A) polymorphisms, and their composite genotype, are related to the 12-month occurrence of low back pain, the number of days with pain, and the number of days with limitations in daily activities due to pain [42]. Carriers of variant allele in IL1-receptor 1 (rs2110726) were less likely to report breast pain prior to surgery in a study with 398 women [43].

### 3.5. IL-4

IL-4 is produced by T cells, mast cells, eosinophilis, and basophiles [140]. It coregulates the inflammatory response by suppressing TNF-*α* and IL-1 expressions [141] and by modulating B cells to undergo Ig isotype switching to IgE [142]. Homozygotes for variant allele (rs2243248) in the gene coding for this anti-inflammatory cytokine were found to be more likely in patients with high degree of depression and pain in a recent study with oncology patients (*n* = 168) and their family caregivers (*n* = 85) [44]. 

### 3.6. IL-6

IL-6 has a role in the regulation of inflammatory response. IL-6 knockout mice have had significantly higher levels of other cytokines in response to endotoxin [143]. In patients with juvenile rheumatoid arthritis, IL-6 genotype −174G/G (rs1800795) was positively correlated with pain [45]. Homozygous carriers of the IL-6 −174C allele required 4.7 times higher dose of opioids for pain relief relative as compared with GG and GC newly diagnosed patients with nonsmall cell lung cancer [37, 38].

### 3.7. IL-8

IL-8 attracts the neutrophiles to the site of infection or injury [144]. Its elevated concentrations are found in various diseases, particularly associated with inflammation, such as rheumatoid arthritis [145]. There are however very few discrepant data on SNP IL8-251T/A (rs4073), where wild-type allele was a predictor for severe pain in 168 Caucasian patients—TT or AT subjects had more than a threefold risk (OR = 3.23, 95%  CI = 1.4, 4.7) for severe pain compared to the AA patients [46]; while in another study, TT homozygotes had the least frequency of severe pain [37].

## 4. Drug Metabolism

The pharmacokinetics of drugs is subject to a large interindividual variability, which is important cause for adverse drug reactions and lack of drug response. In therapy of pain, numerous genetic polymorphisms affecting pharmacokinetics of drugs have been shown to contribute in part to interindividual variability in drug efficacy and safety [146]. The most important drug metabolizing enzymes for pain treatment are cytochromes P450 (P450), UDP-glucuronyltransferases (UGTs), and sulfotransferases (SULTs) [59, 147].  Table 4 summarizes the principal polymorphic DMEs with its most important genetic variants involved in the metabolism of drugs used for chronic pain.  Table 5 shows the drugs used in pain treatment and its major DMEs emphasizing FDA recommendations. An example in which pharmacogenetic testing of DMEs could be clinically relevant is P450 and UDP-glucuronyltransferase [59]. 

Recently, comprehensive and in-depth monography concerning drug metabolism (including impact of genetic polymorphisms) was published [73].

### 4.1. P450

Cytochrome P450 (P450) consists of heme-containing monooxygenase enzymes located on the smooth endoplasmic reticulum membranes of liver hepatocytes and along the mucosal surface of the intestinal tract and several other tissues including kidney, heart, and brain. Research on human P450 polymorphisms began in the 1970s and continues till now [73]. Actual information concerning P450 polymorphisms is compiled on the website http://www.cypalleles.ki.se/. 

### 4.2. CYP2D6

CYP2D6 accounts for 2–5% of the total hepatic P450 enzymes; however, it is involved in the metabolism of 25% of all drugs administered in clinical practice [47]. It is also important for many drugs used in pain and palliative medicine as it is responsible for metabolizing certain analgesics as opioids (codeine, tramadol, hydrocodone, oxycodone), neuroleptics, and antidepressants; see Table 5. CYP2D6 plays an important role not only in the metabolism of exogenous opioids but also in the endogenous morphine synthesis pathway. CYP2D6-metabolized drugs exhibit nonlinear saturable kinetics owing to the low capacity of CYP2D6. The existence of almost 80 CYP2D6 allelic variants is known to lead to phenotype diversity within populations [148]. Approximately 7–10% of people may be classified as poor metabolizers (PM) and 3% as ultrarapid metabolisers (UM) in the Caucasian populations [149, 150]. An example in which pharmacogenetic testing of CYP2D6 is clinically relevant is codeine. Codeine as a prodrug requires *O*-demethylation catalyzed by CYP2D6 to be converted into morphine and become analgesic. This metabolite pathway accounts for 10% of codeine clearance in EMs but is much more pronounced in UMs and far less pronounced in PMs; so PMs suffer from a lack of analgesia, while UMs have been shown to be more likely to experience side effects and have 50% higher plasma concentration of morphine compared to EMs [151]. Codeine as a weak opioid was believed to be a relatively safe analgesic. However, after the death of breastfed neonate through morphine overdose by his mother taking codeine, the safety profile of codeine was reevaluated and FDA published a warning on codeine use in nursing mothers [152]. It is suggested that codeine should be avoided in breastfeeding mothers, who are extensive metabolizers (EMs) or UMs of CYP2D6 [153, 154]. The European Medicines Agency started a review of codeine-containing medicines on October 3, 2012, as well [155]. Young and obese children with history of sleep apnea are also at higher risk of developing severe opioid-related respiratory depression. The adverse outcomes of codeine treatment could be avoided and the safety of pain management could be improved by CYP2D6 genetic testing before prescribing the drug (tramadol, hydrocodone, or oxycodone) or by using alternative analgesics [156]. Another analgesic agent which is metabolized by CYP2D6 and where genetic examination is proposed is tramadol. The main metabolite is *O*-desmethyltramadol; (+)-*O*-desmethyltramadol has 300–400 times greater affinity for *μ*-opioid receptors than tramadol, whereas (−)-*O*-desmethyltramadol mainly inhibits noradrenalin reuptake [157]. Production of *O*-desmethyltramadol through mono-*O*-demethylation is mediated by polymorphic CYP2D6. As consequence, PMs need approximately 30% higher tramadol doses compared to EMs, and UMs are at greater risk to develop adverse effects of tramadol [47, 158]. Genetic testing of variants CYP2D6 is commercially available [159].

### 4.3. CYP2C

The group of CYP2C subfamily consists of four members: CYP2C8, CYP2C9, CYP2C18, and CYP2C19. These enzymes metabolize approximately 20% of clinically available drugs. Their genes are tandemly located at 10q24 and there is a linkage between them. Genetic variants in CYP2C8, CYP2C9, and CYP2C19 have been shown to have clinical consequences. Among pain treatment, NSAIDs represent typical substrates for CYP2C enzymes; however, the relative role of CYP2C enzymes in primary metabolism differs among different NSAIDs.

Common CYP2C8 and CYP2C9 polymorphisms were studied by Blanco et al. in a cross-sectional study, involving 134 NSAID-related bleeding patients and 177 patients receiving NSAID with no adverse effects [160]. Among patients with bleeding after NSAID (CYP2C8/9 substrates), the frequencies of variant alleles carriers versus control patients were 0.50 versus 0.23 (odds ratio (OR); 95% confidence interval (CI) = 3.4; 1.5–7.5; *P* = 0.002), 0.48 versus 0.26 (OR; 95% CI = 2.7; 1.2–5.8; *P* = 0.013), and 0.24 versus 0.20 (OR; 95%  CI = 1.3; 0.5–3.1; *P* = 0.578) for CYP2C8*3, CYP2C9*2, and CYP2C9*3, respectively. These findings were not influenced by gender, age, smoking, or drinking habits. Among bleeding patients receiving NSAID that are not extensively metabolized by CYP2C8/9, no differences in genotypes or allele frequencies were observed as compared to control patients. Similar findings have been shown by other authors; individuals carrying the gene variants CYP2C8*3 (rs11572080; rs10509681), CYP2C9*2 (rs1799853), or CYP2C9*3 (rs1057910) show increased risk of developing acute gastrointestinal bleeding during the use of NSAID that are CYP2C8 or CYP2C9 substrates [161, 162].

### 4.4. CYP2C19

Totally 36 alleles of gene CYP2C19 have been identified and described so far [48]. CYP2C19 is responsible for the metabolism of several clinically important drugs as citalopram, barbiturates, diazepam, and other drugs [52]. The roles of the cytochrome P450 2C19 enzyme and cytochrome P450 2D6 enzyme in citalopram metabolism were studied [163]. The inactive CYP2C19*2 (rs4244285) allele was associated with lower odd ratios for tolerance. The estimated dose adjustments for CYP2C19 poor metabolizers suggest using approximately 60% of the standard dose of citalopram [164]. Also the allelic variant CYP2C19*3 (rs4986893 or rs57081121) influences the total concentration of the active compounds venlafaxine and its active metabolite *O*-desmethylvenlafaxine. Thus, CYP2C19 genotypes (together with CYP2D6 genotypes) should be considered for dose alterations of venlafaxine [165].

### 4.5. CYP2C9

The pharmacokinetics of ibuprofen is strongly related to CYP2C8 and CYP2C9 genotypes. The effect of CYP2C8*3 (rs10509681 or rs11572080) on ibuprofen clearance is prominent; heterozygous and homozygous carriers of this variant allele display clearance reduced to approximately 62% and 10% as compared to individuals lacking any variants within CYP2C8 and CYP2C9 genes [166]. Although initial findings indicated association of CYP2C9*2 (rs1799853) genotypes with ibuprofen clearance, it has been shown that CYP2C9*2 alone, when it is not linked to CYP2C8*3, does not translate into a major impairment of ibuprofen clearance. Clearance values in subjects heterozygous and homozygous for CYP2C9*2 not carrying any other mutations are 96 and 84%, respectively, as compared to individuals lacking any mutations in CYP2C8 and CYP2C9 genes. Individuals carrying CYP2C9*3 (rs1057910) variant alleles display a mean reduction of clearance of *∼*65% and 17% for heterozygous and homozygous individuals, respectively [167].

Studies with tenoxicam have indicated that oral clearance among carriers of CYP2C9*2 and CYP2C9*3 decreases to *∼*70 and 55% [168]; however, efficacy or safety data are not available yet. 

### 4.6. CYP2C8

CYP2C8 comprises 7% of the total hepatic CYP content and plays an important role in the metabolism of a diverse number of exogenous (e.g., NSAIDs, carbamazepine, diltiazem, methadone, morphine, and zopiclone) and endogenous compounds (e.g., arachidonic acid) [55]. A number of common SNPs have been identified including CYP2C8*2 (Ile269Phe and rs11572103), CYP2C8*3 (linked polymorphism Arg139Lys and Lys399Arg, rs10509681, or rs11572080), and CYP2C8*4 (Ile264Met and rs1058930). One of the drugs implicated as CYP2C8 substrate is methadone. *In vitro*, CYP2C8 was shown to metabolize both the R- and S-enantiomers of methadone, with a greater selectivity for R-enantiomer [169]. Considering that the R-enantiomer is the more pharmacologically active form *in vivo*, the potential influence of CYP2C8 polymorphism on the metabolism of the R-enantiomer may be clinically significant and warrants further studies. 

Allelic variants of CYP2C8, UGT2B7, and ABCC2, which may predispose for the formation and accumulation of reactive diclofenac metabolites, are associated with diclofenac hepatotoxicity [55]. Daly et al. showed that UGT2B7*2 allele (rs7439366) was more common in patients with diclofenac-induced hepatotoxicity when compared with hospital controls (OR, 8.5, *P* = 0.03) or healthy controls (OR, 7.7, *P* = 0.03). Further, the ABCC2 C-24T (rs717620) variant was more common in patients with hepatotoxicity compared with hospital (OR 5.0, *P* = 0.005) and healthy controls (OR 6.3, *P* = 0.0002). Haplotype distributions for CYP2C8 were different between patients and hospital controls (*P* = 0.04).

### 4.7. CYP3A4

CYP3A4, coded by the gene located on chromosome 7q21.1, is involved in the oxidation of the largest range of substrates of all the CYPs. CYP3A4 plays a role in the metabolism of some opioids as fentanyl, oxycodone, and methadone along with the other CYPs [148]. There is little conclusive information about the importance of genetic variation in the CYP3A pathway, but some studies of postmortem forensic toxicology propose pharmacogenomics of CYP3A4 as a kind of molecular autopsy in the analysis of pain-medications-related deaths. In studies of fentanyl-, oxycodone- or methadone-related deaths, the PM status was a clear risk factor [170–172].

### 4.8. UDP-Glucuronyltransferase

Glucuronidation is an important pathway of human metabolism that leads to the formation of water soluble glucuronides. The substrates for glucuronidation include both endogenous substances, such as bilirubin, steroid hormones, and bile acids, and exogenous substances such as morphine, antidepressants, or nonsteroidal anti-inflammatory drugs. The human genome codes for at least 19 different UDP-glucuronosyltransferases (UGTs) classified within three subfamilies, UGT1A, 2A, and 2B [173]. Genetic polymorphisms have been reported in virtually every UGT family member and many of them have potential clinical consequences. For example, morphine undergoes extensive metabolism by glucuronidation to form morphine-3-glucuronide and morphine-6-glucuronide which possess significant analgesic activity. The ability to glucuronidate morphine varies substantially between individuals. The major enzyme responsible for glucuronidation of morphine is UGT2B7. The variant homozygotes for T/T802 (Y/Y268) displayed the strongest catalyzing abilities toward morphine, and this genotype has been considered as a one of the possible causes of interindividual variability in therapeutic response to morphine [174]. 

A small cross-sectional study observed faster *in vivo* conjugation of salicylic acid in patients genotyped UGT1A6*2/*2 (rs2070959) than in the wild-type carriers. The faster conjugation may subsequently influence the therapeutic response to aspirin [175].

## 5. Drug Transport

There are many families of transporters and some of them are known to be interacting with pathways of pain-transporting analgesics, prostaglandins [22]. So far the most known and best studied transporter is P-glycoprotein (Pgp), the first-studied member of ATP-binding cassette (ABC) superfamily. In humans, Pgp consists of two isoforms with 78% amino acid homology. Overexpression of isoform I (ABCB1) in cancer cells was linked with resistance to multiple drugs, hence the name for this transporter multidrug resistance protein 1 (MDR1). Isoform II (MDR2/ABCB4) transports phosphatidylcholine into the bile and is not involved in drug transport [176, 177].

Summary of known implications of genetic variability in genes for drug and neurotransmitter transporters is given in Tables 6 and 7, respectively.

### 5.1. MDR1/ABCB1

Pgp is known to show extremely broad substrate specificity, including peptides, steroids, therapeutic drug from very large and complex ones as paclitaxel [178] to relatively simple as phenytoin, opioids, and other analgesics [179] or even ions [180–186]. Substrates are often, but not always (e.g., colchicine), amphipathic and relatively hydrophobic. Planar aromatic ring and tertiary amino group were also proposed as required structure elements, but many peptides do not have them, and yet they are substrates of Pgp [187].

In addition to this broad substrate specificity, Pgp is also very abundant in the body as it can be found in most tissues [188], although in significantly larger amount on the apical surface of the endothelial cells lining the small intestine, colon, kidney, adrenal gland, bile ductules, thus in the tissues with excretory (or absorptive) function in general. In addition, also in cells with “barrier” function, that is, cells in blood brain [189], blood testis [190], blood mammary tissue [191], blood inner ear barrier [192], and in placenta [193], protecting respective tissues (or fetus [194]) from toxins in the blood. Recent studies show that this may not be the only one physiological function of Pgp or even the crucial one. It seems that Pgp is involved in the inhibition of apoptosis induced by a number of factors as tumor necrosis factor and ultraviolet and gamma radiations [195]. Further, it was shown that blocking Pgp by antibodies induced apoptosis of activated lymphocytes in peripheral blood, and MDR1/ABCB1 seems to regulate even stem cells [196]. Secretion of various cytokines (interleukin 2 and 4, interferon *γ*) is mediated by Pgp [197].

Pgp is a product of the ATP-binding cassette, subfamily B (MDR/TAP), member 1 gene (*ABCB1*), gene of 209617 bp with 29 exons of total length 4872 bp located on chromosome 7q21.12. There are 1425 known SNPs to date with average. distance of 161 bp; 46 of them are nonsynonymous: two of them in introns and 46 in coding sequence of exons [198]. The synonymous SNPs and SNPs in promoter regions could influence the expression level of MDR1/ABCB1, for protein activity, that is, its substrate binding, ATP hydrolysis and folding, are most important probably the nonsynonymous SNPs [195]. Because of the important role of Pgp in drug disposition, it seems as a fair presumption that such SNPs could have clinical importance in drug pharmacokinetics and, by extension, in pharmacodynamics (and even direct impact on PD in tumor cells with overexpression of MDR1/ABCB1). Campa et al. found that variability of pain relief in 145 patients on morphine treatment was significantly associated with SNP C3435T (rs1045642). The association was stronger; when C3435T was combined with A80G in OPRM1 SNPs were taken into account [27]. There were significant C3435T- dependent differences in morphine concentrations in cerebrospinal fluid (CSF) with the highest levels in CSF in TT carriers of SNP C3435T and the highest morphine-6- and 3-glucuronide concentrations in CSF in wild-type homozygotes [81]. Response to morphine was dependent on SNP C3435T in children in a recent study using Faces Pain Scale (FPS). Scores >6 were more frequent in 11 checks during 24 hours after orthopedic or abdominal surgery in carriers for the wild-type alleles (adjusted risk ratio = 4.5; 95% confidence interval (CI), 1.5–13.4; corrected CI for multiple comparisons, 0.98–20.55) [83]. 

For fentanyl, variability in suppression of respiratory rate (significant only for C3435T and diplotype) and need for oxygen (increased in carriers of 1236T (rs1128503) and 3435T alleles, *P* = 0.0847) were observed, and significant differences in the level of respiratory suppression were found in patients with linked 3435T and 2677T (rs2032582) alleles [84]. 

The atypical opioid tramadol was proposed to be a subject of P-glycoprotein-dependent transport, as there were significant differences in its *C*_max⁡_ and borderline significant differences in AUC_0–24_ amongst different genotypes for MDR1/ABCB1 in CYP2D6 poor metabolizers [85]. Conversely, no significant differences among MDR1/ABCB1 subgroups with regards of pain difference, drug consumption, reporting of adverse reactions, need for rescue analgesic medication, or verbal description of pain were observed [158]. For oxycodone, strong associations between variant alleles 3435T and 2677A and less adverse drug reactions and better analgesic effect and variant 2677A were found in study with 33 healthy volunteers and experimental pain [86]. 

Apart from opioids, Pgp is believed to transport several antiepileptic drugs (AED), for example, lamotrigine, gabapentin, topiramate, valproic acid, and carbamazepine and its ketoanalog oxcarbazepine, although there is no consensus about this and studies with positive [199–201] or negative results [202–204] may be found. The efflux may play facilitatory role in refractory epilepsy [205–207], although contradictory results are also available [208]. These agents are widely used in the treatment of neuropathic pain. There are only pharmacogenetic studies in epilepsy in association with MDR1/ABCB1 polymorphisms, but their results could give some guidance about the impact of SNPs in MDR1/ABCB1 in the treatment of neuropathic pain.

In the case of lamotrigine, homozygotes for the C allele in C1236T have had significantly higher lamotrigine dose corrected concentrations (0.068 *μ*mol · l^−1^ · mg^−1^) than subjects with CT or TT (0.053 *μ*mol · l^−1^ · mg^−1^). Furthermore, 1236C-2677G-3435C carriers have had higher lamotrigine concentrations than 1236T-2677G-3435T carriers (*P* < 0.001), followed by 1236T-2677T-3435C carriers (*P* < 0.001) [87]. 

Change in gabapentin's disposition due to 2677T/A MDR1/ABCB1 alleles was less pronounced resulting only in trend toward higher values of the absorptive phase characterized by the AUC (0-1 h) and AUC (0–1.5 h) [88]. 

SNP C3435T did not influence disposition of valproic acid as its serum concentrations of the patients with CT, TT and CC genotypes were 72.92 ± 20.55, 80.47 ± 14.01, and 68.29 ± 12.17 *μ*g/mL, respectively, and there was no significant difference [209]. 

Carbamazepine appears to be subject of Pgp transport, its clearance was associated with rs1128503, being significantly lower in subjects with alleles CC versus CT + TT [89]. The median total carbamazepine plasma levels were the lowest in CC (20 *μ*mol/L) homozygotes followed by CT (23 *μ*mol/L) and TT (29 *μ*mol/L) carriers of SNP 3435 [90]. However, Meng et al. suggested that ABCB1 3435TT is associated with decreased plasma carbamazepine levels in Chinese patients with epilepsy [91]. On the other hand, Hung et al. did not find any difference in carbamazepine levels among genotype groups for SNPs C1236T, G2677T/A, and C3435T in MDR1/ABCB1 [210]. Moreover, some studies did not found any association between C3435T and epilepsy treatment response [211] or with dosage [212].

Speaking of neuropathic pain, even tricyclic antidepressant drugs, such as amitriptyline, nortriptyline, desipramine, and SNRI (venlafaxine and duloxetine), are being used for treating this condition, all of which are subject to the Pgp mediated efflux [213–219], which was implicated to be associated with refractory depression [220]. 

In knockout mice, venlafaxine and its three demethylated metabolites reached significantly higher concentrations than in wild-type mice [92–94]. Similar results were obtained with trimipramine [96] and amitriptyline [97, 98].

Case study was reported in which SNP rs2232583 in MDR1/ABCB1 apparently resulted in excessive plasma levels of venlafaxine and its metabolite desmethylvenlafaxine in the patient [95]. 

### 5.2. MRP

Multidrug resistance-associated proteins (MRP) also belong to the ABC transporters family, subfamily ABCC, which consists of 12 members, nine of them are MRPs. Similar to MDR1/ABCB1, they utilize ATP but share only 24% of amino acid sequence homology [221]. Thus, there are distinct substrate specificity, inhibitors, and tissue distribution as compared to P-glycoprotein. Mainly, three of MRPs are known to interact with the pathways of pain: MRP2/ABCC2, which transfers diclofenac's metabolites [55, 99], MRP3/ABCC3, which was shown to transfer morphine [101], and diclofenac's glucuronides [99], and MRP4/ABCC4, which transports most prostaglandins [222] (even proposed as prostanoid export pump [223, 224]) and acetylsalicylic acid [225, 226]. 

As for MRP2/ABCC2, genetic variability could lead to impaired clearance of diclofenac and its glucuronides and hence to hepatotoxicity, as it was shown with knockout mouse [99]. One SNP (C24T, rs717620) in 5′-untranslated region was associated with decreased mRNA expression [227, 228], although many other SNPs have been found [229, 230]. Allele 24T was found to be more prevalent in patients with hepatotoxicity as compared with patients taking diclofenac for 0.3–20 years (*n* = 48) without hepatotoxicity (OR 5.0, *P* = 0.005) and healthy controls (OR 6.3, *P* = 0.0002) [55].

Carbamazepine appears to be subject of ABCC2 transport, as its clearance was significantly higher in subjects with alleles AA + AG versus GG in rs2273697 and rs4148386 [89]. Ufer et al. strengthen this assumption since in their study carriers of the ABCC2 1249G>A (rs934847) variant were more frequently classified as responders to treatment and this impact was even more pronounced among 64 patients receiving carbamazepine or oxcarbazepine [231]. 

Involvement of MRP4/ABCC4 in export of prostanoids could have significant clinical implication for nociception and analgesia, as was shown in MRP4/ABCC4 knockout mice in study by Lin et al., where disruption of MRP4/ABCC4 resulted in decreased pain responsiveness [102]. Further, recent study associated SNP rs9524885 in MRP4/ABCC4 (as well as rs2756109 in MRP2/ABCC2) with pain in nonsmall-cell lung cancer patients [100].

### 5.3. SLC22A6

Another drug transporter known to transport analgesics, particularly NSAIDs [232], is SLC22A6, human organic anion transporter 1. However, the clinical relevance for chronic pain treatment of its variation has not been clarified yet. 

### 5.4. SLCO1B1

SLCO1B1, also known as OATP2, was shown to transport opioid peptides across blood brain barrier [233] therefore, it is possible that genetic variability may have influence on the pain perception. This has not been clinically assessed yet, but immunofluorescence microscopy and uptake measurements were used to study localization and transport properties. The polymorphisms SLC21A6*1b and SLC21A6*4 have been associated with altered transport of cholyltaurine and 17 beta-glucuronosyl estradiol [234]. 

### 5.5. 5-HTT

Genetic polymorphism of serotonin transporter (5-HTT) has also been associated with alteration of pain pathways. For example, study with 43 healthy volunteers found that subjects with the triallelic 5-HTTLPR genotype coding for low 5-HTT expression gained better analgesic effect of remifentanil compared to those homozygous for the 5-HTTLPR LA allele, although the baseline sensitivity to heat pain was not affected by the triallelic 5-HTTLPR polymorphism [103]. Low 5-HTT-expressing group compared to the high 5-HTT-expressing group exhibited significantly increased pressure pain and heat-pain thresholds [104], while contradicting results have been described by Aoki et al. [235]. Association between inferred low 5-HTT expression and elevated thresholds to thermal pain was found in 44 healthy nondepressed individuals [105]. Similarly, homozygotes for variable-number tandem-repeat polymorphism 2.10 suffered less from temporomandibular joint pain and dysfunction [106, 107]. Two recent meta-analyses found that non-STin2.12 alleles possess protective effect compared to STin2.12 alleles, respectively, 10/12 and 10/10 genotypes compared to the 12/12 genotype against migraine among populations of European descent [108], while no overall association between the SLC6A4 5-HTTLPR polymorphism and migraine among Europeans and Asians was found, though gender and migraine aura status may have modifying roles among Europeans [236].

Repeat variation polymorphism in 5-HTT gene consists of a short (s) variation of 14 repeats of a sequence and a long (l) variation of 16 repeats. Subjects with irritable-bowel syndrome with s/s genotype for 5-HTT have suffered more often from abdominal pain than l/s and l/l [109]. A significantly higher frequency of the s/s genotype of the serotonin transporter promoter region was found in fibromyalgia patients (31%) compared with healthy controls (16%) in study with 62 patients and 110 healthy controls [110], but this was later contradicted in different study with 53 mentally healthy subset of fibromyalgia patients and 60 healthy controls [237].

### 5.6. Others

Dopamine transporter (DAT, SLC6A3) and glutamate transporter protein excitatory amino acid transporter 2 (SLC1A2, EAAT2) polymorphisms have also been reported to affect pain perception. Allele DAT*10 was significantly underrepresented in patients with chronic daily headache associated with drug abuse when compared with the migraine-without-aura group [111] and A allele carriers of −181 A/C in EAAT2 polymorphism used significantly more analgesics than non-A carriers in migraine patients with chronic daily headache [112].

## 6. Conclusion

There is number of candidate genes whose genetic variability may translate in either individual variation of chronic pain perception or treatment response. The clinical data from pharmacogenetic studies is still very limited and heterogenous as a result of various methodologies used in different studies, generally small sample sizes and heterogenous patient populations. Therefore, there is still a need for further clarifications of the clinical importance for all these findings, but the recent research in the field that encompasses larger studies and larger-scale genome perspectives may bring more promising findings in the future. 

## Figures and Tables

**Table 1:** Overview of recently released (2010–2012) studies assessing the influence of various gene polymorphisms on pain perception in humans.

Gene	Refrence	Polymorphisms	Populations	Results
	Hocking et al., 2010 [1]	Totally 11 SNPs	*n* = 8572, 1958 British birth cohort (83% Caucasians)	No associations of either chronic widespread pain or pain status with COMT genotypes or haplotypes
	Finan et al., 2010 [2]	rs4680	*n* = 46 female fibromyalgia patients (93.0% Caucasians)	Individuals with met/met genotype experienced a greater decline in positive effect on days when pain was elevated more than did either val/met or val/val individuals,* COMT *genotype contributing 1% of variance over and above the effect of pain on PA
	Fijal et al., 2010 [3]	rs6269, rs4633, rs4818, rs4680, and haplotypes	*n* = 159/93 female/male Caucasians with major depressive disorder	Associations between a haplotype created using rs6269, rs4633, rs4818, and rs4680, and the proportion of female patients with “Pain While Awake” and “Overall Pain” at baseline. No association was found in males
	Fernandez-de-las-Penas et al., 2011 [4]	rs4680	*n* = 70 children with chronic tension type headache, *n* = 70 healthy children	Children with chronic tension type headache (CTTH) met/met genotype-longer headache history compared with met/val (*P* = 0.001) or val/val (*P* = 0.002), children with CTTH, met/met genotype showed lower pressure pain test score over upper trapezius and temporalis muscles than children with CTTH with met/val or val/val genotype.
COMT	Barbosa et al., 2012 [5]	rs4680 and rs4818	*n* = 112 fibromyalgia patients *n* = 110 healthy individuals	SNP rs4818, the frequency of variant genotype CC was 73.21 and 39.09% for patients with FS and controls, respectively, Fibromyalgia Impact Questionnaire score was higher in patients with the homozygous variant genotype for SNPs rs4680 (87.92 points) and rs4818 (86.14 points)
	Loggia et al., 2011 [6]	rs4680	*n* = 54 healthy subjects	met/met subjects exhibited stronger pain-related fMRI signals than val/val in several brain structures, including the periaqueductal gray matter, lingual gyrus, cerebellum, hippocampal formation, and precuneus
	Dai et al., 2010 [7]	rs6269, rs4633, rs4818, rs4680, and haplotypes	*n* = 69 patients with low back pain who underwent an intervention	rs4633 T allele—greater improvement in ODI (Oswestry disability index) score 1 year after surgery ATCA haplotype-APS-average pain sensitivity (9.3% in the study population)—greater improvement in ODI. The greatest mean improvement in ODI-ATCA-homozygotes
	Omair et al., 2012 [8]	rs4633, rs4680, rs4818, rs6269, rs2097603, and haplotypes	*N* = 93 patients with low back pain	Association of rs4633 and rs4680 with posttreatment improvement in VAS, for better improvement among heterozygous patients compared to the homozygous ones, no association was observed for the analysis of the common haplotypes
	Martinez-Jauand et al., 2013 [9]	rs6269, rs4633, rs4818 rs4680, and haplotypes	*N* = 113 fibromyalgia patients *n* = 65 healthy controls	Fibromyalgia individuals with the met/met genotype (Val158Met SNP) or the high- and average-pain sensitivity-associated haplotypes showed higher sensitivity to thermal and pressure pain stimuli than patients carrying the LPS haplotype or val alleles (Val158Met SNP)

OPRM1	Klepstad et al., 2004 [10]	A118G (rs1799971)	*N* = 99 Caucasians	Brief pain inventory average pain scores higher in AG heterozygotes
	Olsen et al., 2012 [11]	A118G	*N* = 258 patients with lumbar disc herniation and sciatic pain, Caucasians	∗/G women had 2.3 times as much pain as the ∗/G men 12 months after the disc herniation, while A/A women and A/A men had almost exactly the same recovery rate
	Menon et al., 2012 [12]	A118G	*n* = 153 chronic migraine females, Caucasians	G118 allele carriers were more likely to be high pain sufferers compared to homozygous carriers of the A118 allele (OR = 3.125, 95% CI = 1.41, 6.93, *P* = 0.0037)
	Finan et al., 2010 [2]	A118G	*n* = 46 female patients with fibromyalgia 93.0% Caucasians	Patients with an 118G allele reported higher positive affect score across diary days than those homozygous for 118A
	Janicki et al., 2006 [13]	A118G	*n* = 121 chronic, non-cancer pain patients, *n* = 101 opioid-naive subjects with acute postoperative pain, Caucasians	The frequency of 118G is significantly lower in the subjects with chronic pain than in the group with acute postoperative pain—0.079 versus 0.158; *P* = 0.009

GCH1	Heddini et al., 2012 [14]	rs8007267 rs3783641 rs10483639	*n* = 98 women with provoked vestibulodynia, healthy controls *n* = 102	Significant interaction effect of GCH1 gene polymorphism and hormonal contraceptive therapy on coital pain among patients with current treatment (*n* = 36)

TRPV1	Carreno et al., 2012 [15]	rs222741	*n* = 1040 cases—Caucasians, 1037 controls	Association of rs222741 with the overall migraine group

SCN9A	Reeder et al., 2013 [16]	rs6746030	*n* = 53 biopsy specimens, *n* = 26 control specimens	AA or AG genotypes were present in 39.6% patients with cystitis/bladder pain syndrome—statistically significant difference compared with the controls: 11.5%

KCNS1	Costigan et al., 2010 [17]	rs734784	*n* = 1359 six independent cohorts	rs734784 significantly associated with higher pain scores in five of six independent patient cohorts, lumbar back pain with disc herniation—association with greater pain outcome in homozygote patients. The combined *P* value for pain association in all six cohorts

CACNG2	Nissenbaum et al., 2010 [18]	Totally 12 SNPs	*n* = 549 breast cancer patients: *n* = 215 control group *n* = 334	rs4820242, rs2284015, rs2284017, rs2284018, and rs1883988 showed significant association with chronic pain

ADR*β*2	Diatchenko et al., 2006 [19]	Totally 8 SNPs and their haplotypes H1, H2, and H3	*n* = 181 cohort of females (Caucasians)	H1/H2 and/or H1/H3—lowest temporomandibular disorder incidence—1.3%, H1/H1 elevated risk of developing temporomandibular disorder (RR = 8.0, 95% CI = 1.2–52.2, 99% CI = 0.815–79.7), H3/H3, H2/H3, and H2/H3 H1 elevated risk of developing temporomandibular disorder (RR = 11.3, 95% CI = 1.95–67.9, and 99% CI = 1.38–102
	Hocking et al., 2010 [1]	rs12654778 and rs1042713	*n* = 8572, 1958 British birth cohort (82.6% Caucasians)	ADRB2 SNPs rs12654778 and rs1042713 were associated either with chronic widespread pain alone or with pain status

HTR2A	Nicholl et al., 2011 [20]	Totally 47 SNPs	*n* = 164, control group *n* = 172	rs12584920T (T/∗, T/T) increased likelihood of having chronic widespread pain (OR) = 1.64, 95% confidence interval (95% CI) = 1.01–2.60 (*P* = 0.03) in the discovery cohort, and OR = 1.46, 95% CI = 1.07–2.00 (*P* = 0.018) in the validation cohort, similar association between rs17289394 and the maximum number of pain sites reported in both cohorts

VAS: visual analogue scale, OR: odds ratio, RR: relative risk, CI: confidence interval, SNP: single-nucleotide polymorphism, fMRI: functional magnetic resonance. GCH1: GTP cyclohydrolase 1, the rate limiting enzyme in the biosynthesis of tetrahydrobiopterin is an essential cofactor in the synthesis of serotonin, nitric oxide, and catecholamines. These neurotransmitters are known to modulate pain perception. TRPV1: transient receptor potential cation channel, subfamily V, member 1, acts as an integrator of multiple painful stimuli in chronic pain conditions. SCN9A: sodium channel, voltage-gated, type IX, alpha subunit encodes the voltage-gated sodium channel. Homozygotes with 2 loss-of-function alleles are congenitally indifferent to pain without other neurological deficit. KCNS1: voltage-gated potassium channel 1. CACNG2: calcium channel, voltage-dependent, gamma-subunit 2, encodes the gamma-2 transmembrane AMPA receptor protein (TARP) stargazin. This protein is known to be involved in the modulation of the ion channel function of glutamatergic AMPA receptors. ADRB2-beta2-adrenergic receptor is a target for epinephrine. HTR2A: 5-hydroxytryptamine (serotonin) receptor 2A. P2X7: cAMP responsive element binding protein 1.

**Table 2:** Trials assessing the influence of gene polymorphisms associated with neurotransmission on drug response in humans.

Gene	Refernces	Drugs	Polymorphisms	Populations	Results
	Laugsand et al., 2011 [21]	Opioids (morphine, oxycodone, fentanyl, others)	rs4680, rs4633	*n* = 1579 cancer patients (European Caucasians) from the cohort of [22]	C allele of rs165722, the T allele of rs4633 and the G allele of rs4680 had less nausea/vomiting
	Reyes-Gibby et al., 2007 [23]	Morphine	rs4680	*n* = 207 cancer	Carriers of val/val and val/met genotype required 63% and 23%, respectively, higher morphine dose compared to carriers of met/met genotype
	Lötsch et al., 2009 [24]	Various opioids		*n* = 352 patients with chronic pain of various origin	No association
COMT	Klepstad et al., 2011 [22]	Morphine (*n* = 830), oxycodone (*n* = 446), fentanyl (*n* = 699), or other opioids (*n* = 234)	112 SNPs in the 25 candidate genes including OPRM1 A118G	*n* = 2294 cancer patients, European Caucasians	None of SNPs in the candidate genes *OPRM1, OPRD1, OPRK1, ARRB2, GNAZ, HINT1, Stat6, ABCB1, COMT, HRH1, ADRA2A, MC1R, TACR1, GCH1, DRD2, DRD3, * *HTR3A, HTR3B, HTR2A, HTR3C, * *HTR3D, HTR3E, HTR1,* or *CNR1* showed significant associations with opioid dose
	Rakvåg et al., 2008 [25]	Morphine	11 SNP and haplotypes, including rs4680, rs4633 not included	*n* = 197 Caucasian cancer patient cohort receiving oral morphine treatment for cancer pain	The most frequent haplotype (34.5% rs2075507, rs737866, rs7287550R, rs5746849, rs740603, rs6269, rs2239393, rs4818, rs4680 (Val158Met) rs174699, rs165728 GACAAAACATT) associated with lower morphine doses, with a reduction factor of 0.71
	Ross et al., 2008 [26]	Morphine	13 SNPs, rs4818 not included	*n* = 228 cancer patients on morphine	Haplotype in intron 1 (AATTGAAATAATT) and 4873G genotype (10% is strongly associated with somnolence), hallucinations and confusion after treatment with morphine (protective effect). ABCB1 genotypes and haplotypes investigated in the study as well allele 21/2677G and 12/1236C associated with somnolence, hallucinations, and confusion after treatment with morphine (protective effect)

	Reyes-Gibby et al., 2007 [23]	Morphine	A118G	*n* = 207 cancer patients, Caucasians	GG genotype required 93% higher morphine dose compared to carriers of AA genotypes (*P* = 0.012)
	Klepstad et al., 2004 [10]	Morphine	A118G	*n* = 99 cancer patients, Caucasians	No association with the intensities of symptoms such as fatigue, nausea and vomiting, dyspnea, sleep disturbance, loss of appetite, and constipation were similar between the three cohorts, The serum concentrations of morphine, M6G, and M3G were higher in patients homozygous for the 118G allele
	Campa et al., 2008 [27]	Morphine	A118G	*n* = 145 Italian Caucasians	Significant association of pain relief after treatment with morphine with the allele. The association improved with the combination of the allele and polymorphism in ABCB1 detection of three groups: strong responders, responders, and nonresponders, sensitivity →→ 100%, specificity > 70%
	Lötsch et al., 2009 [24]	Various opioids	A118G	*n* = 352 patients with chronic pain of various origin	Tendency towards increased pain in dose-dependent manner with the *μ*-opioid receptor variant 118G. Daily opioid doses significantly decreased in a gene dose-dependent manner with the P-glycoprotein variant ABCB1 3435C>T
OPRM1	Liu and Wang 2012 [28]	Acetaminophen/ tramadol	A118G	*n* = 96 patients with adenocarcinoma of the colon or rectum (*n* = 84), or stomach (*n* = 12) who developed oxaliplatin-induced painful neuropathy	The requirement for rescue analgesia higher for patients with G allele, AA genotype-better analgesic effect than G allele variants (AG or GG genotypes). Pretreatment and posttreatment VAS scores for patients with G allele variants were 3.1 and 2.6, respectively; for patients with AA genotype, pretreatment and posttreatment VAS scores were 3.0 and 0.9
	Janicki et al., 2006 [13]	Morphine	A118G	*n* = 121 chronic, noncancer pain patients, Caucasians	The mean opioid dose is significantly larger in the homozygous carriers of the wild-type 118A allele when compared with the carriers of the variant allele
	Klepstad et al., 2011 [22]	Morphine (*n* = 830), oxycodone (*n* = 446), fentanyl (*n* = 699), or other opioids (*n* = 234)	112 SNPs in the 25 candidate genes including OPRM1 A118G	*n* = 2294 cancer patients, European Caucasians	None of SNPs in the candidate genes *OPRM1, OPRD1, OPRK1, ARRB2, GNAZ, HINT1, Stat6, ABCB1, COMT, HRH1, ADRA2A, * *MC1R, TACR1, GCH1, DRD2, DRD3, * *HTR3A, HTR3B, HTR2A, HTR3C, * *HTR3D, HTR3E, HTR1,* or *CNR1* showed significant associations with opioid dose
	Droney et al., 2013 [29]	Morphine	A118G	*n* = 264 cancer patients taking oral morphine	Genetic factors only accounted for 12% of variability in residual pain on morphine and 3% of variability in central side effects

CREB1	Nishizawa et al., 2012 [30]	Opioids			rs2952768 was associated with more analgesic requirements, and consistent results were obtained in patients who underwent abdominal surgery

HTR3B	Laugsand et al., 2011 [21]	Opioids (morphine, oxycodone, fentanyl, and others)	rs1176744, rs3782025 rs1672717	*n* = 1579 cancer patients (European Caucasians)	G allele of rs1176744, the T allele of rs3782025, and the T allele of rs1672717 were associated with less nausea/vomiting

CHRM3	Laugsand et al., 2011 [21]	Opioids (morphine, oxycodone, fentanyl, and others)	rs10802789 rs685550	*n* = 1579 cancer patients (European Caucasians)	T allele of rs10802789 associated with more nausea/vomiting

KCNJ6	Lötsch et al., 2010 [31]	Methadone	rs2070995	*n* = 352 opioid-treated chronic pain patients	The daily methadone substitution doses during the first therapy year were larger in the rs2070995 AA genotype (*n* = 4, 119.7 ± 49.6 mg/day) than in other rs2070995 genotypes (77.5 ± 26.2 mg/day, P = 0.003)

DRD4	Ho et al., 2008 [32]	Heroin	−521C/T	*n* = 43 current heroin uses, 66 controls	TT control subjects had lower pain threshold versus CC/CT controls and versus TT addicts

HTR2C	Brash-Andersen et al., 2011 [33]	Escitalopram	rs6318	*n* = 34 patients with peripheral neuropathic pain	rs6318 (Cys23Ser) in the HTR2C gene showed significant association with treatment response in men, with 75% carrying the C allele being responders. The same tendency was seen in women

VAS: visual analogue scale. CREB1: cAMP responsive element binding protein 1 encodes a transcription factor, a member of the leucine zipper family of DNA binding proteins. HTR3B: 5-hydroxytryptamine (serotonin) receptor 3B encodes subunit B of the type 3 receptor for serotonin (neurotransmitter, hormone, and mitogen). Activation of the receptor leads to fast depolarizing responses in neurons. Pentaheteromeric complex with subunit A (HTR3A) displays the full functional features of this receptor. HTR2C encodes the 2C subtype of serotonin receptor. CHRM3: the muscarinic cholinergic receptor 3, G-protein-coupled receptor controls smooth muscle contraction, and its stimulation increases secretion of glandular tissue. KCNJ6: gene for potassium inwardly rectifying channels, subfamily J, member 6 (Kir3.2, GIRK2). This G channel is important for opioid receptor transmission and is involved in opioid effects on postsynaptic inhibition [34]. DRD4: dopamine receptor D4 belongs to the dopamine receptor D2-like family, which mediates reward and reinforcement effects (e.g., of heroin) [35].

**Table 3:** Impact of genetic variability in genes for proinflammatory cytokines.

Gene product	Genetic variability	Effect	Reference
TNFR2	TNFR2(−/−) mice	Attenuated hyperalgesia	[36]

TNF *α*	A allele in −308G/A (rs1800629)	Higher pain severity	[37–39]
	G allele in −308G/A (rs1800629)	Anti-inflammatory treatment success with phytotheraphy	[40]

LTA	Variant allele in rs5275	Lower pain scores	[41]

COX 2	CC in rs5275	Lower risk of severe pain	[39]
	G allele in rs5277	Higher pain scores	[41]

IL-1*α*	C889-T (rs1800587)	Pain intensity	
IL-1*β*	C3954-T (rs1143634)	Occurrence of low back pain, the number of days with pain, and the number of days with limitations in daily activities due to pain	[42]
IL-1RN	G1812-A		

IL1-receptor 1	Variant allele in rs2110726	Less frequent breast pain	[43]

IL-4	Varant allele in rs2243248	More frequent pain	[44]

IL-6	−174G/G (rs1800795)	Pain	[45]
	−174C/C (rs1800795)	Higher opioid dosage	[37, 38]

IL-8	T allele in 251T/A (rs4073)	More frequent severe pain	[46]
	251T/T (rs4073)	Least frequent severe pain	[37]

**Table 4:** The principal polymorphic DMEs involved in the metabolism of drugs used for chronic pain.

Enzymes	Important gene variants	Influenced drug group	Proven effect on PK or efficacy/safety in clinical trials	References
CYP2D6	CYP2D6*1-wt CYP2D6*3 2549A>del CYP2D6*4 1846G>A CYP2D6*5 CYP2D6*6 1707T>del MxN CYP2D6*10	TCA Opioids SSRI	Opioids (codeine, tramadol, hydrocodone, and oxycodone), TCA (amitriptyline, nortriptyline, imipramine, and desipramine), and SSRI (fluoxetine, paroxetine, and citalopram)	[47, 48]

CYP2C9	CYP2C9*1-wt CYP2C9*2 (Cys144Arg) CYP2C9*3 (Leu359Iso) CYP2C9*5	NSAIDs SSRIs SNRIs	Coxibs (celecoxib)	[48–50]

CYP2C19	CYP2C19*1-wt CYP2C19*2 CYP2C19*3 CYP2C19*17-Ums	NSAID Antidepressants	SSRI (citalopram)	[48, 51, 52]

CYP2C8	CYP2C8*1-wt CYP2C8*2 (Ile269Phe) CYP2C8*3 (Arg139Lys, Lys399Arg)	NSAID	NSAID (ibuprofen and diclofenac)	[48, 53–55]

CYP3A4	CYP3A4*1 (2023G>A) CYP3A4*2 CYP3A4*10 CYP3A4*17	Opioids	Opioids (methadone and fentanyl)	[48, 56–58]

UGT1A6	UGT1A6*1 UGT1A6*2	NSAIDs, anticonvulsants	NSAID (acetylsalicylic acid)	[59, 60]

UGT2B7	UGT2B7*2 (802C>T, H268Y), 161C>T	NSAID Opioids Anticonvulsants	Opioids (morphine) and anticonvulsants (lamotrigine and valproic acid)	[61–65]

UGT1A1	UGT1A1*28	Paracetamol Opioids	Paracetamol	[62, 66]

SULT1A1	SULT1A1*2 (G638A; Arg213His) SULT1A1*3 (A667; Met223Val)	Paracetamol	Paracetamol	[67]

**Table 5:** Drugs used in pain treatment and its major DMEs emphazing FDA recommendations for genetic testing [73–75].

Drug class	Drug	Major enzymes			FDA drug labels including pharmacogenetics information
		CYPs	UGBTs	SULTs	
Analgesic/ antipyretics	Paracetamol	3A4, 2E1, 2A6, 1A2, 2D6, 2C19, 2C9, 2E1, 2A6	1A6, 1A9	1A1	In combination with tramadol CYP2D6

NSAIDs	Ibuprofen	2C9, 2C8, 3A4, 2C19, 2C9, 2C8, 3A4, 2C19	2B7, 1A9, 1A3, 2B4		
	Diclofenac	2C9, 2C8, 3A4, 2C19	2B7, 2B4, 1A3, 1A9		
	Naproxen	2C9, 1A2			
	Ketoprofen		2B7		
	Meloxicam	2C9, 3A4			

Coxibs	Celecoxib	2C9, 3A4			CYP2C9
	Etoricoxib	3A4, 2C9, 2D6, 1A2, 2C19			

TCAs	Amitriptyline	2C19, 2C8, 2C9, 1A2, 2D6, 3B6, 3A4	1A3, 1A4		CYP2D6
	Nortiyptyline	2D6, 3A4			CYP2D6
	Imipramine	2D6, 2C19, 1A2			CYP2D6
	Desipramine	2D6			CYP2D6

SNRIs	Duloxetine	2D6, 1A2			
	Venlafaxine	2D6, 2C19, 2C9			CYP2D6

	Fluoxetine	2C9, 3A4, 2D6, 2C19, 1A2			In combination with olanzapine, and CYP2D6
SSRIs	Paroxetine	2D6			CYP2D6
	Citalopram	3A4, 2C19, 2D6			CYPs 2C19, and 2D6

Antiepileptics	Carbamazepine	3A4, 2C8	2B7		
	Valproate		2B7, 1A6, 1A9		

Opioids	Buprenorphine	3A4, 2C8	1A1, 2B7, 1A3		
	Codeine	2D6, 3A4	2B7, 2B4		CYP2D6
	Dihydrocodeine	2D6, 3A4	2B7		
	Morphine	3A4, 2C8	2B7, 1A8, 1A1, 1A3, 1A10, 1A6, 1A1		
	Oxycodone	2D6, 3A4	2B7		
	Pethidine	3A4, 2B6, 2C19			
	Tilidine	3A			
	Tramadol	3A4, 2B6			In combination with paracetamol CYP2D6

**Table 6:** Impact of genetic variability in genes for drug transporters.

Transporter	Drug	Genetic variability	Effect	Reference
		61 (rs9282564), 1199 (rs2229109), 1236 (rs1128503), 2677 (rs2032582), and 3435 (rs1045642)	Lower dosage	[76]
		C1236 (rs1128503)	Higher dosage	[77]
	Methadone	61A (rs9282564) : 1199G (rs2229109) : 1236C (rs1128503) : 2677T (rs2032582) : 3435T (rs1045642)	Higher dosage	[78]
		61A (rs9282564) : 1199G (rs2229109) : 1236C (rs1128503) : 2677T (rs2032582) : 3435T (rs1045642)	Lower through concentrations	[78]
		61G (rs9282564) and 3435T (rs1045642)	Lower through concentrations	[79]
		3435T (rs1045642)	Higher dosage	[80]
		C3435T (rs1045642)	Pain relief	[27]
		3435TT (rs1045642)	Higher CSF concentrations	[81]
	Morphine	CC3435 (rs1045642)	Higher CSF concentrations of morphine glucuronides	[81]
		GG2677 (rs2032582) and CC3435 (rs1045642)	Fewer side effects	[82]
		G2677 (rs2032582) and C3435 (rs1045642)	Vomiting	[61]
		3435T (rs1045642)	Less frequent pain scores >6	[83]
	Fentanyl	3435T (rs1045642)	Suppression of respiratory rate	[84]
MDR1/ABCB1	Tramadol	3435TT (rs1045642)	Higher *C*_max⁡_	[85]
	Oxycodone	3435T, 2677A (rs2032582)	Fewer side effects	[86]
		2677A (rs2032582)	Better analgesic activity	[86]
	Lamotrigine	C1236 (rs1128503)	Higher dose corrected concentrations	[87]
	Gabapentin	2677T/A (rs2032582)	Trend towards higher AUC(0–1.5 h)	[88]
		CC1236 (rs1128503)	Significantly lower clearance	[89]
	Carbamazepine	CC3435 (rs1045642)	Lowest plasma levels	[90]
		3435TT (rs1045642)	Decreased plasma levels	[91]
	Venlafaxine	MDR1/ABCB1(−/−) mice	Higher plasma levels	[92–94]
	Venlafaxine's metabolites	MDR1/ABCB1(−/−) mice	Higher plasma levels	
	Venlafaxine	TT in rs2232583	Higher plasma levels	[95]
	Trimipramine	MDR1/ABCB1(−/−) mice	Higher plasma levels	[96]
	Amitriptyline	MDR1/ABCB1(−/−) mice	Higher plasma levels	[97, 98]

MRP2/ABCC2	Diclofenac	MRP2/ABCC2(−/−) mice	Impaired clearance	[99]
	Diclofenac	24T (rs717620)	Hepatotoxicity	[55]

	Carbamazepine	AA + AG in rs2273697	Higher clearance	
		AA + AG in rs4148386	Higher clearance	[89]
	—	rs2756109	Pain	[100]

MRP3/ABCC3	Morphine	MRP3/ABCC3(−/−) mice	Increase in plasma levels of its glucuronides	[101]

MRP4/ABCC4	—	MRP4/ABCC4(−/−) mice	Decreased pain responsiveness	[102]
	—	rs9524885	Pain	[100]

**Table 7:** Impact of genetic variability in genes for transporters of neurotransmitters.

Transporter	Drug	Genetic variability	Effect	Reference
5-HTT	Remifentanil	Triallelic 5-HTTLPR	Better analgesic effect	[103]
	—	Low 5-HTT-expressing	Higher pain thresholds	[104, 105]
		Tandem-repeat polymorphism 2.10	Lesser temporomandibular joint pain and dysfunction	[106, 107]
		10/12 and 10/10 STin2.12 alleles	Protective effect against migraine	[108]
		14/14 sequence repeats	Higher frequency of abdominal pain	[109]
			More frequent in fibromyalgia patients	[110]
DAT	—	DAT*10	More frequent in migraine-without-aura-group	[111]
EAAT2	Analgesics	A allele in −181A/C	Higher usage	[112]

## Abbreviations

CYP: Cytochromes P450
DME: Drug-metabolizing enzyme
EM: Extensive metabolizer
FDA: Food and drug administration
IM: Intermediate metabolizer
NSAID: Nonsteroidal anti-inflammatory drug
PK: Pharmacokinetics
PM: Poor metabolizer
SNP: Single-nucleotide polymorphism
SNRI: Serotonin-norepinephrine reuptake inhibitor
SSRI: Selective serotonin reuptake inhibitor
SULT: Sulfotransferase
TCA: Tricyclic antidepressant
UGT: UDP-glucuronyltransferase
UM: Ultrarapid metabolizer.
